# Determinants of household demand for bed nets in a rural area of southern Mozambique

**DOI:** 10.1186/1475-2875-8-132

**Published:** 2009-06-15

**Authors:** Claire Chase, Elisa Sicuri, Charfudin Sacoor, Delino Nhalungo, Ariel Nhacolo, Pedro L Alonso, Clara Menéndez

**Affiliations:** 1 Harvard School of Public Health, Department of Global Health and Population, Boston, MA, USA; 2 Centre de Recerca en Salut Internacional de Barcelona (CRESIB), Hospital Clínic/Institut d'Investigacions Biomèdiques August Pi i Sunyer, Universitat de Barcelona, Rosselló 132, E-08036 Barcelona, Spain; 3 Centro de Investigação em Saúde de Manhiça (CISM), Maputo, Mozambique

## Abstract

**Background:**

A key to making insecticide-treated nets (ITNs) a long-term, sustainable solution to the spread of malaria is understanding what drives their purchase and use. Few studies have analysed the determinants of demand for bed nets for malaria prevention at the household level, and in particular, how demand for nets compares with demand for other mosquito prevention methods.

**Methods:**

This study uses a household survey to assess the determinants of demand for bed nets in an area of endemic malaria transmission in rural, southern Mozambique. The study looks at willingness to pay (WTP) for bed nets, net ownership, usage, and past purchase behaviour, alongside expenditure and frequency of use of alternate methods for malaria prevention.

**Results:**

While overall net ownership in the sample is low, the evidence fails to suggest that poorer households are less likely to own bed nets, when controlling for covariates, nor does the likelihood of receiving a free net depend on socioeconomic status (SES). Formal schooling and market knowledge seem to indicate higher average willingness to pay, while use of alternate methods for malaria prevention, and receipt of Indoor Residual Spraying (IRS) are found to decrease demand for bed nets.

**Conclusion:**

For long-term sustainability of ITNs to be realized, results suggest that either full or partial subsidies may be necessary in some contexts to encourage households to obtain and use nets. Given the possible substitution effects of combined malaria control interventions, and the danger of not taking into consideration household preferences for malaria prevention, successful malaria control campaigns should invest a portion of their funds towards educating recipients of IRS and users of other preventive methods on the importance of net use even in the absence of mosquitoes.

## Background

Malaria is the principal cause of morbidity and mortality in Mozambique, responsible for 40% of outpatient visits and 60% of pediatric hospital admissions [1]. The Mozambican Ministry of Health estimates that malaria prevalence rates among children under five are as high as 90% in some areas, and that malaria accounts for 30% of registered paediatric hospital deaths. Insecticide-treated nets (ITNs) are regarded as one of the most effective prevention methods available to decrease this burden, yet they continue to remain under-utilized despite widespread efforts to introduce and sustain their use. At current usage levels, ITNs have failed to achieve their full public health impact [2]. Much of the debate surrounding ITN usage centers on the question of how to achieve full-scale implementation and take-up. Reliance on markets to distribute nets can lead to inequalities in net ownership, as many of those who stand to benefit are too poor to purchase the nets at market price [3]. Market-led distribution has been further hampered by supply side financial and logistical constraints to market expansion in remote regions [4]. Alternatively, handing out millions of nets for free is both time and cost-prohibitive, and while it has been shown to achieve the greatest coverage rates [5], free distribution may not necessarily translate into higher usage, leading to inefficiencies as well. Donor-funded malaria control campaigns have long employed partially-subsidized nets as a means to cover some of the costs associated with procurement and delivery [6,7]. However, cost-sharing programmes are often criticized for generating inequities in distribution and net use [3,8]. Furthermore, cost-sharing programmes ignore the potential community benefits of ITNs [2,9,10], which make public subsidies designed to encourage public consumption more appealing [11].

While there is still no consensus on the best way to deliver ITNs [12], and cost-effectiveness measures have shown both strategies to be attractive [13,14], one thing remains clear: rapid scale up is necessary in order to reach the 2010 Abuja target of halving malaria deaths [15]. In many settings this implies the use of large campaigns to distribute nets for free to target populations of pregnant women and children under five, especially the poor. Crucially, however, many of these campaigns are now complemented by social marketing efforts and targeted subsidies designed to popularize healthy behavior and stir demand for ITNs, even in the presence of free distribution. Vouchers distributed to women through ante-natal clinics (ANC) can be a way to bridge the gap between the rural poor, typically lacking access to nets, and formal markets. The vouchers lower the out-of-pocket expense to the consumer while at the same time encouraging market growth of ITNs [16]. Other programmes make use of market segmentation to offer differently priced nets to different target groups [17]. A key to making such interventions more effective and sustainable in the long term is understanding what drives the purchase and use of ITNs, as well as other malaria prevention methods.

Few studies have analysed the determinants of demand for bed nets for malaria prevention at the household level, and in particular, how demand for nets compares with demand for other malaria prevention methods [18-20]. To develop successful net promotion campaigns, policymakers need to be aware of both the characteristics of net-buying households, as well as household preferences in the prevention of malaria. This study uses contingent valuation to assess willingness-to-pay (WTP) for bed nets in a rural area of southern Mozambique, where malaria is endemic. While WTP for ITNs has been examined in previous literature, estimates here are combined here with more objective measures of a household's preferences via household net ownership, usage, and past purchase behaviour, alongside expenditure and frequency of usage of alternate prevention methods. The study looks at a subset of households who have received Indoor Residual Spraying (IRS) as part of a government programme to examine the potential substitution effects of combined malaria control interventions such as IRS and ITNs.

## Methods

### Study design

The study was carried out in Manhiça District, a rural area of southern Mozambique, where the Manhiça Health Research Center (Centro de Investigação em Saúde de Manhiça – CISM) has been running a continuous Demographic Surveillance System (DSS) since 1996. Malaria, caused principally by *Plasmodium falciparum*, is endemic year round in Manhiça District, but is particularly prevalent during the warm, rainy season from October to March [21]. The detailed characteristics of the study site have been described in detail elsewhere [22].

Households from the CISM DSS study area were invited to participate in a survey with interviews taking place during routine demographic census rounds over a period of three weeks in August 2007. Fieldworkers were trained to administer the questionnaire in both Portuguese and Changana, the language spoken by the Changana people who inhabit the area. The questionnaire was administered to the head of household or a representative over the age of 18, with interviews taking place in the respondent's home. The survey data were supplemented with demographic surveillance data routinely collected in the study area by matching household and respondent IDs collected in the questionnaire to those contained in the demographic database. Supplementary data obtained in the demographic files include household characteristics such as number of structures in the housing compound, type of material used in housing construction, type of fuel used for cooking, presence and type of latrine, and presence of a kitchen. In addition, age, sex, level of education, and number of household inhabitants were taken from the DSS. Household fumigation by IRS and the number of nets owned by the household were also indicated in the DSS. The data were also linked to hospital records containing malaria cases detected and diagnosed through the formal health system, and used to calculate malaria prevalence by enumeration area.

A sample of 1,008 households was calculated from a population of 18,000 households, assuming a 95% confidence level, maximum variance, and a margin of error of 3%. Fieldworkers were provided with surveys to administer alongside the routine census. A total of 1,069 surveys were carried out, of which 1,046 could be matched to their demographic data from the DSS. A further 63 surveys were unusable due to missing values (n = 26) or because the respondent was under 18 (n = 37). A total of 983 (91.9%) household observations were used in the final analysis. No households were reported to have refused participation in the survey.

### Study tool and contingent evaluation procedure

A short questionnaire was developed to elicit household WTP for bed nets, bed net market knowledge, and bed net ownership and past purchase behaviour. The questionnaire was developed after a literature review of similar studies using the contingent valuation method to assess WTP for health 'goods' [23-25], and was piloted in 15 households over a period of three days. The contingent valuation method is frequently used in health and environmental economics to place a monetary value on non-market goods in cases where individual purchase behaviour cannot be directly observed.

While bed nets can be bought through private sector markets in Manhiça District, they are more often distributed for free or obtained through quasi-private markets at subsidized prices. There are no data available in the study area on bed net purchases, and the low frequency at which households purchase nets makes it virtually impossible to observe these market transactions. Moreover, the objective of the study was not to set a market price for bed nets, as is frequently the goal of contingent valuation, but rather to gather information on and describe the determinants of demand.

There are several commonly used methods to elicit WTP, but studies comparing these methods have found dichotomous choice and bidding approaches to be the most reliable [26,27]. In this study, respondents were first asked their hypothetical WTP for a net using a binary response method of elicitation. The survey followed up by asking respondents whether they would pay 100 MZN ($4) for a net. Those who responded no, were asked to provide the maximum amount they would be willing to pay. The initial price is often staggered when using the binary with follow-up (BWFU) method in order to generate a demand curve for the goods or service; however this study offered the nets at a single price of 100 MZN, the market price of conventional nets at the time. Indeed, those who were willing to pay the stated price of 100 MZN may actually be willing to pay more. While both indicators of WTP in this study are hypothetical, in that they fail to observe actual purchase behaviour, in the analysis that follows, hypothetical WTP refers to the binary response part of the question, while mean stated, or average, WTP refers to the monetary amount the respondent indicates they are willing to pay. All prices were provided in Mozambique Meticals (MZN), and are expressed here in 2008 US Dollars using the Financial Times currency converter [28].

### Description of variables used in the analysis

#### Socio-economic status

Socio-economic status (SES) is measured using principal components analysis (PCA) on several household characteristics captured in the DSS census. These characteristics are type of material used to construct the primary residence, number of sleeping divisions in the household, and number of household members per sleeping room. Additional household characteristics such as type of cooking fuel used in household and type of latrine were excluded from the PCA since there was very little variation among households in the study area (90.4% use wood for cooking and 89.7% of households have a primitive latrine), and including these variables would not help distinguish between household SES. Table 1 shows the mean, standard deviation and factor score, or weight, for each variable used in the index. A negative factor score, such as that for dwellings constructed of cane and high occupancy per room are associated with lower SES. For instance, poorer households in the district are generally constructed of cane, while less poor households may be a mix of cane and concrete. Few households outside of the town center are constructed of concrete. The other two variables, number of sleeping divisions and household members per sleeping room are an indication of household and family size. PCA is able to explain 40.5% of the variation in household characteristics in the first principal component, generally understood to be the measure of SES [29]. For ease of interpretation, the socioeconomic index is rescaled from 0 to 1 and households are categorized into 5 SES quintiles of close to equal proportion, with the lowest SES quintile (Q1) representing the poorest households and the highest quintile (Q5) representing the least poor. The quintile categories are unevenly distributed due to limited variability in household characteristics making it difficult to distinguish between some households. The proportion of households falling into each quintile are: Q1 = 22.48% Q2 = 20.55% Q3 = 17.5% Q4 = 19.53% Q5 = 19.94%.

**Table 1:** Variables used in principle component analysis

Variable	Mean	Std. Dev.	Min	Max	Factor score
House made of cane	0.65	0.48	0	1	-0.66
House mix of cane and modern	0.27	0.44	0	1	0.61
House modern construction	0.08	0.27	0	1	0.16
Number sleeping divisions	2.07	1.04	1	8	0.35
Number persons per room	3.37	1.78	0.5	16	-0.19

#### Consumer behaviour

Consumer behaviour is a construct measured using responses collected on market knowledge and past purchase behaviour. Market knowledge indicates that respondents know where nets are sold and how much they cost, while past purchase behaviour is indicated by asking respondents if they had ever purchased a net in the past, how much they paid for it, and if they had ever received a net for free. Respondents were not asked when the purchases took place.

#### Malaria prevention behaviour

Respondents were asked to recall what prevention measures they took during the previous 'mosquito season' to prevent mosquitoes. The specific wording of this question was agreed upon following discussions with anthropologists in Manhiça regarding community understanding of malaria transmission. In-depth interviews conducted in the study area as part of a separate study indicate that people have a basic understanding of malaria and its symptoms. They also associate malaria with mosquitoes and are aware of various methods to prevent illness, including bed nets [30]. In the analysis that follows, the mosquito season is referred to as the rainy season and is understood to mean the period from October to March when the majority of malaria transmission occurs. Other prevention measures included mosquito coils, repellent spray, prophylaxis (sulphadoxine-pyrimethamine), bed nets, and traditional methods such as the burning of herbs. They were then asked to recall their expenditure over the entire season on this method and to indicate the household frequency of use. It was not feasible to ask respondents to provide expenditures and usage over a two week recall period, since the questionnaire was administered more than four months after the bulk of mosquito prevention activity would typically occur. Given this longer recall period expenditure estimates are likely subject to recall bias that may underestimate total expenditure [31].

### Statistical analysis

The relationship between SES and malaria prevention behaviour was assessed by comparing responses to questions about expenditure on prevention measures during the previous rainy season, net ownership, and previous net purchases. To examine inequalities in demand for nets, responses were compared across SES quintiles on hypothetical household WTP and mean stated WTP. Inequalities in market knowledge were assessed by comparing the proportion of households who knew where nets were sold and how much they cost. To determine whether these outcomes varied significantly across SES quintiles chi-square tests were used.

Tobit regression [32] was used to investigate variations and determinants of WTP for bed nets. Because the 'amount willing to pay' variable was restricted to values between 0 MZN and 100 MZN a two-limit Tobit regression model was used. Other determinants of net ownership, purchase behaviour, and hypothetical WTP were analyzed using multiple regression analysis. SES was included as a categorical variable (SES quintile) in baseline regressions, however models employing SES as a continuous index and household characteristics as proxies for SES status were tested but not found to impact the results. Logistic regression was used to explain variation in hypothetical WTP, net ownership and usage, previous purchase, and receipt of free net. Finally, ordinary-least squares (OLS) was used to explain variation in the amount paid for net and previous expenditure on malaria prevention.

## Results

### Sample description

Household ownership of nets (Table 2) and SES quintile do show a positive correlation: of the 365 (37.1%) households owning a net, the poorest SES quintile accounts for the lowest proportion of net ownership at 15.9%. Those in the highest SES quintile were most likely to have previously purchased a net (23.8%), while the poorest quintile was most likely to have received a net for free (23.5%), however the difference is not significant for receipt of a free net. A relatively high proportion of least poor had also received a net for free in the past, possibly reflecting improved access to services or better access to knowledge regarding the availability of free nets. Overall 34.9% (n = 344) of those surveyed had previously received a net for free, which was larger than the proportion who had purchased a net in the past (28.7%; n = 282) at a mean purchase price of 80.5 MZN ($3.30). For those respondents who did not own a net at the time of the survey, the most common reason provided was cost, with 54.9% saying that nets were too expensive. Another 27% did not own a net because their previous net had torn or been ripped.

**Table 2:** Household ownership of nets and malaria cases, by income quintile

Quintile	No. households owning a net		No. nets per household	No. households bought net		No. households received free net		Avg. malaria case rate per1000 hospital person years (2007)
	N	%	N	N	%	N	%	
Q1 (most poor)	58	15.9	0.34	48	17.0	81	23.5	120.6
Q2 (very poor)	70	19.2	0.49	61	21.6	71	20.6	109.8
Q3 (poor)	69	18.9	0.56	46	16.3	60	17.4	101.5
Q4 (less poor)	73	20.0	0.57	60	21.3	54	15.7	87.6
Q5 (least poor)	95	26.0	0.86	67	23.8	78	22.7	75.3
Total	365	100	0.56	282	100	344	100	99.6
Chi2	23.3***			9.3*		6.2		

*, *** statistically significant relationship between the two variables

Rates of malaria cases diagnosed through the formal health system appear to follow a clear SES gradient with average cases among the poorest recorded as 120.6 per 1,000 hospital person years, while those in the least poor SES quintile recorded an average of 75.3 cases per 1,000 hospital person years (Table 2). Since these figures reflect cases diagnosed through the formal health system the low rates reported for the highest SES group could well indicate improved access to anti-malarials and less frequent visits to the hospital, as opposed to lower disease exposure.

Willingness-to-pay and household expenditure on malaria prevention are presented in Table 3. Households in the least poor SES quintile spent an average of 73.0 MZN ($3.00) on malaria prevention during the previous rainy season, 77.8% more than was spent in the lowest SES quintile. As hypothesized, the average stated WTP appears to increase along the SES gradient, with 19.0% of the lowest SES quintile willing to pay an average of 66.9 MZN ($2.74). Among the least poor those hypothetically willing to pay increases slightly to 22.1% with an average WTP of 77.0 MZN ($3.16).

**Table 3:** Household willingness to pay (wtp) and expenditure on malaria prevention, by income quintile

Quintile	No. households hypothetically WTP		No. households hypothetically WTP 100 mzn		Avg WTP(mzn)	Avg. prevention expenditure in rainy season (mzn)
	N	%	N	%		
Q1 (most poor)	170	22.0	100	19.0	66.9	41.0
Q2 (very poor)	151	19.5	114	21.7	72.5	57.6
Q3 (poor)	141	18.2	87	16.5	71.4	49.3
Q4 (less poor)	149	19.3	109	20.7	75.3	60.0
Q5 (least poor)	162	21.0	116	22.1	77.0	73.0
Total	773	100	526	100	72.5	55.9
Chi2	5.3		10.7**			

** statistically significant relationship between the two variables

### Net ownership and usage

The results of net ownership and usage are shown in Table 4. 38.3% of respondents reported sleeping under a mosquito net during the previous rainy season and of these 94.1% reported that they slept under the net every night, indicating that while the proportion who use a net is still low, those who do use a net, tend to use it consistently. A high 86.3% of respondents reported using an alternate method other than a bed net for prevention of mosquitoes during the previous rainy season. There is some evidence of a substitution effect between available malaria prevention methods, as households who reported use of alternate methods were significantly less likely to own a net, and less likely to report using a net. Households from areas with higher malaria case rates were also less likely to own a net, however these data do not enable us to determine whether this is caused by a lack of net ownership. Separately, respondents were asked whether they had purchased a net in the past. Households in higher SES quintiles were more likely to have purchased a net (column 6), with a unit increase in SES quintile associated with about a third higher likelihood. Those with formal schooling were 2.8 times as likely as those with no education to have purchased a net (column 6).

**Table 4:** Net ownership and usage^1^

Variable	Owns net (logit)	Robust SE	Uses net (logit)	Robust SE	Bought net (logit)	Robust SE
Formal schooling	0.72	0.26	0.96	0.33	2.80**	1.16
SES quintile (Q1-Q5)	1.05	0.09	0.99	0.08	1.30***	0.13
Household received IRS	1.21	0.18	0.84	0.12	1.62***	0.28
Uses alternate method	0.22***	0.05	n/a	n/a	0.36***	0.08
Malaria cases	0.05***	0.04	0.10***	0.08	0.05***	0.05
No. of observations	980		980		980	

^1^ Odds ratios reported

Covariates: In addition to those shown, all regressions control for age, sex, occupation, number household members, interaction term between

SES quintile and formal schooling, knowledge of where nets are sold and price, head of household respondent, and child under 5 present in the household

*Significantly different from 0 at 90 percent confidence

**Significantly different from 0 at 95 percent confidence

***Significantly different from 0 at 99 percent confidence

### Willingness-to-pay for bed nets

Output showing the results of multiple regression analysis on the three outcome variables for the entire sample: stated WTP, hypothetical WTP, and average household expenditure on mosquito prevention are shown in Table 5. Stated WTP is significantly higher for young educated respondents (column 2), with formal schooling contributing an additional $0.80 to average WTP (p < 0.001). The increase is roughly equivalent to 20% of the market price of a bed net. Likewise, movement between SES quintiles increases WTP (p = 0.002), although the effect itself is small, making up just 3% of the market price of a bed net. Despite predicting higher WTP, it is found that respondents with both formal schooling and higher SES scores show no measurable increase in WTP.

**Table 5:** Household willingness to pay (wtp)^1^

Variable	Stated WTP (tobit)^2;3^	SE	Hypothetical WTP(logit)^4^	Robust SE	Previous exp. (OLS)	Robust SE
Age	-2.28***	0.79	0.87**	0.06	-2.75	2.10
Formal schooling	19.67***	4.96	0.92	0.36	5.32	12.15
SES quintile (Q1-Q5)	3.32***	1.09	1.00	0.09	3.37	2.74
Quintile*formal schooling	-4.19***	1.42	1.07	0.13	0.49	4.13
Household received IRS	-4.28**	2.15	1.40*	0.25	7.82	6.49
Uses alternate method	-5.04*	2.86	1.51*	0.38	n/a	n/a
Malaria cases	-3.21	12.23	11.37**	10.86	-12.65	35.86
Constant	n/a	n/a	n/a	n/a	13.62	19.41
No. of observations	980		980		980	

^1^ Stated WTP and Previous expenditure expressed in Mozambican Meticals 24 MZN = 1 USD

^2^ Marginal effects after tobit y = E(wtpj0 <wtp < 100) = 76.88

^3^ Tobit censored observations: 52 left-censored at wtp < = 0; 404 uncensored; 524 right-censored at wtp > = 100

^4^ Odds ratios reported

Covariates: In addition to those shown, all regressions control for sex, occupation, number household members, number of nets owned, knowledge of

where nets are sold and price, receipt of free net in the past, frequency of net use, head of household respondent, and child under 5 present in the household

*Significantly different from 0 at 90 percent confidence

**Significantly different from 0 at 95 percent confidence

***Significantly different from 0 at 99 percent confidence

Substitution of alternate methods of malaria prevention appears to be a significant determinant of household demand for nets, with respondents reporting the use of alternate methods and respondents from households that had received IRS both stating a lower average WTP (p = 0.078 and 0.046 respectively). In terms of magnitude, households that had received IRS were willing to pay $0.17 less on average for a net, controlling for covariates. The magnitude is similar for households who report using an alternate method of prevention during the previous rainy season, such as coils, sprays, or traditional methods. Conversely, the odds of hypothetical WTP (column 4) are higher and significant at the 10% level for households that have received IRS, which seems to indicate the two may be complements. In other words, although households appear place some value on malaria prevention methods such as bed nets, they prefer to spend their money on other methods.

Table 6 shows the results of multiple regression analysis on hypothetical WTP and stated WTP for the sub-sample of households who did not own a net (n = 616; 62.6%). These households are arguably a more interesting subset, as they represent the population who stand to benefit the most from targeted bed net promotion. Education is again found to have a positive and significant effect on WTP, averaging $0.84, as is knowledge of where nets are sold. There is strong evidence that households where a child under five is present have a higher average WTP (p = 0.004), possibly indicating the higher value placed on child health, or awareness of the acute vulnerability of children to malaria. Contrary to popular opinion and potentially problematic for some social marketing programmes, which target messages to these groups, is the evidence that females and heads of household both state less WTP on average. To know whether the results are different for mothers of small children, an interaction term between female respondent and presence of a child under five in the household was tested, but found to be insignificant. While the magnitude of the effect is small in both cases, these findings seem to indicate that household decision making authority is an important determinant of the demand for nets.

**Table 6:** Willingness to pay in households without net^1^

Variable	Stated WTP (tobit)^2;3^	SE	Hypothetical WTP (logit)^4^	Robust SE
Child under 5 in household	9.76***	3.39	1.56*	0.42
Female	-6.21**	2.95	0.81	0.22
Head of household	-12.57***	3.26	0.71	0.18
Formal schooling	20.61***	5.81	0.94	0.46
SES quintile (Q1-Q5)	4.37***	1.32	0.93	0.10
Household received IRS	-5.4**	2.76	1.59**	0.36
Knows where nets sold	8.52***	3.11	2.18***	0.60
Knows cost of net	2.26	3.3	3.28***	1.08
Received free net	-6.11*	3.43	1.65*	0.44
Uses alternate method	-1.02	5.03	2.01*	0.79
Malaria cases	-15.46	15.27	11.48**	13.84
Constant	n/a	n/a	0.16	0.76
No. of observations	616		616	

^1^ Stated WTP expressed in Mozambican Meticals 24 MZN = 1 USD

^2^ Marginal effects after tobit y = E(wtpj0 <wtp < 100) = 77.46

^3^ Tobit censored observations: 35 left-censored at wtp < = 0; 248 uncensored; 333 right-censored at wtp > = 100

^4^ Odds ratios reported

Covariates: In addition to those shown, all regressions control for age, occupation, number household members, receipt of free net in the past,

interaction term between SES quintile and formal schooling

*Significantly different from 0 at 90 percent confidence

**Significantly different from 0 at 95 percent confidence

***Significantly different from 0 at 99 percent confidence

## Discussion

The findings presented here indicate that WTP for bed nets in an area of rural, southern Mozambique is highly dependent on formal schooling, market knowledge of bed nets, and use of alternate malaria prevention methods, including IRS. While SES is found to have a significant effect on WTP, with respondents of higher SES willing to pay more on average, the magnitude of the effect is small, leaving little evidence to suggest that WTP differs widely by SES. There is no significant difference in formal schooling between SES quintiles (p = 0.132), so the effect of education on WTP would in all likelihood transcend SES status. Moreover, there is no evidence that poorer households are less likely to own bed nets when controlling for covariates, nor does the likelihood of receiving a free net depend on SES quintile. Only the likelihood of buying a net is significantly higher for those in the topmost SES quintile. There are multiple explanations as to why this may be. It could be that the poorest are less likely to buy nets because they are less 'able to pay' for the nets. In this study, SES is used as a proxy for ability-to-pay, under the normal assumption that higher SES households have a higher ability-to-pay, and is controlled for in all the models. While this assumption is subject to limitations, it is found that previous expenditure, perhaps a better indicator of ability-to-pay, was higher, although not significantly different, for the upper SES quintiles (Table 5). The fact that this figure is positive, however, may indicate something about the capital investment required to purchase a net as opposed to other prevention methods, which cost less but require more frequent purchase. In other words, given the high price of nets they may require households to plan and save in advance in order to purchase, as opposed to coils and sprays which can be found much cheaper. To put the cost of a bed net in perspective for this population, it is useful to evaluate income and expenditure in Mozambique. While Gross Net Income (GNI) in 2006 was estimated at US$310 [33], monthly per capita expenditures for Maputo Province where the District of Manhiça is located are among the highest in the country, at an estimated US$19.56 per month (2002 figures). However, 70.4% of these expenditures are on the essentials of food, housing, and cooking fuel, leaving little income left over for other necessities. Indeed, spending on health accounts for just US$0.75 per household per month. There are large disparities in spending by quintile, with individuals in the poorest quintile spending on average just US$3.61 per month versus US$35.14 by the richest. Furthermore, while health accounts for 1.5% of aggregate spending among the least poor, it is only 0.9% of total spending for the poorest [34]. Another possible explanation as to why the poorest SES quintile state lower WTP, and are less likely to buy a net, may be a lack of information regarding the health benefits of nets and their availability through public or private channels. Indeed, respondents with market knowledge state a higher WTP. Similarly formal schooling, which is found to be positively correlated with demand for nets, may provide some of the information necessary to trigger demand.

Another notable finding is the negative correlation between stated WTP and the use of alternate methods for malaria prevention, including IRS. The relationship is not surprising: if IRS is successful in ridding households of mosquitoes there is little incentive to use a bed net. Additionally, if households are provided information on the effectiveness of IRS they may be persuaded to discontinue sleeping under a bed net -a plausibly rational behaviour change. If malaria control programmes rely on a combination of IRS and ITNs to achieve lower malaria prevalence, such substitution effects could be problematic. In order to achieve maximum effectiveness, IRS must be consistently applied over several years in order to avoid resurgence of the vector in an otherwise unprotected population. According to personal communication with the National Malaria Control Program, routine IRS began in the end of 2005 and is ongoing in Manhiça District with spraying taking place annually just prior to the rainy season. As part of the routine census, households were asked if and when they had received IRS, and according to these data the last documented spraying took place in March 2007. There are several methodological challenges with the current study, which are common to many studies assessing WTP, particularly in resource-poor settings. While SES was not found to be a substantial driver of WTP, the lack of a complete index comprising household amenities and possessions is a weakness of the SES classification used in this study. More generally, while PCA is widely used in the determination of SES, it is unable to measure fiuctuations in income or income shocks [35]. In an economy such as that of Manhiça, which is highly dependent on subsistence agriculture, income is likely to fiuctuate seasonally making it difficult for households to plan future expenditures. Additionally, since the outcome of interest, WTP, depends on income availability, the absence of expenditure data is potentially problematic for its measurement.

Another key methodological challenge is measuring WTP. Contingent valuation is only able to measure an individual's stated preference. A more objective measure of WTP would analyse actual market transactions for the purchase of bed nets. These data are available for a subset of the sample who had previously purchased a net, however they are subject to recall bias and lack a clearly defined purchase window since respondents were not asked when the purchase took place. Since observing this behaviour ex-ante is difficult, in order to overcome the obstacle some studies have observed purchase behaviour of the study sample by offering nets for sale a short time after administering the WTP survey. Furthermore, this study was done during the cool, dry season. A follow-up study looking at WTP during the rainy season, when the need for nets to combat mosquitoes has reached a peak, may shed some light on the validity of WTP estimates. One hypothesis for the fiuctuation in WTP may be linked to the availability of income. That is, when respondents have the cash available, they may state a higher WTP. Another hypothesis could be that the economic circumstances of households in this area lead them to discount the future at a higher rate, whereby consumption is focused on present need with little attention to perceived future need. A study which looks at WTP during the rainy season would provide valuable information on the reliability of WTP responses, and contribute to evidence on demand sensitivity to level of risk.

This study looks at demographics, market knowledge, and product availability as determinants of WTP for bed nets. However, there may be other factors not measured in this study, which contribute to a household's WTP. For instance, the presence of NGOs in a community or the presence of large malaria prevention campaigns may both influence WTP. Campaigns may help disseminate knowledge and promote the use of nets, which may make the purchase of nets more attractive to households. Free or heavily subsidised nets that are part of these campaigns may displace a regular market for nets, thereby decreasing WTP. While the evidence presented here shows that households are WTP less on average for a net if they had received one for free in the past, they also appear to place a higher value on the nets than households who had not received a free net. Since distribution of free nets in the area is erratic, and there is no evidence that such programmes have a long standing presence in the study area, it is not likely that household are 'holding out' for a free net. Indeed, market saturation of heavily subsidised or free nets would inevitably lead to lower WTP as households discover the real price of a net, whether free or below market price.

## Conclusion

This study uses a household survey to assess the determinants of demand for bed nets in a rural area of southern Mozambique. The study looks at WTP for bed nets, net ownership, usage, and past purchase behaviour, alongside expenditure and frequency of usage of alternate methods for malaria prevention. Overall net ownership in the study area is low, and in households where nets are present, respondents report obtaining them through both public and private channels. While receipt of a free net is not found to differ significantly by SES quintile, previous net purchase is higher among respondents from households of higher SES. The most likely reason provided for not owning a net is cost, and, indeed, stated WTP averages less than three-quarters of the market price of a net. In order to increase coverage of this life-saving intervention, and to achieve long-term sustainability of ITNs in rural areas of Africa, for the most part lacking access to sophisticated markets, these results suggest that either full or partial subsidies may be necessary to encourage households to obtain and use nets. This is particularly the case for regions with similar demographic and socioeconomic characteristics to the study area, and for areas which have not benefited substantially from large donor-funded malaria prevention campaigns. The study findings also draw attention to the potential substitution effects of combined malaria control interventions, and the danger of not taking into consideration household preferences for mosquito prevention, and ultimately malaria control. Since the combined use of IRS and ITNs is now considered a core strategy of malaria prevention through mosquito control, campaigns advocating this strategy should invest a portion of their funds towards educating recipients of IRS and users of other mosquito control methods on the importance of net use even in the absence of mosquitoes.

## Competing interests

The authors declare that they have no competing interests.

## Authors' contributions

CC and CM conceived of the study, led its design and coordination, and helped to draft the manuscript. ES helped to analyse data. ES, CS, DN, AN, and PA participated in the design and coordination of the study and reviewed and edited all previous drafts. All authors read and approved the final manuscript.
